# Enriching the Study Population for Ischemic Stroke Therapeutic Trials Using a Machine Learning Algorithm

**DOI:** 10.3389/fneur.2021.784250

**Published:** 2022-01-25

**Authors:** Jenish Maharjan, Yasha Ektefaie, Logan Ryan, Samson Mataraso, Gina Barnes, Sepideh Shokouhi, Abigail Green-Saxena, Jacob Calvert, Qingqing Mao, Ritankar Das

**Affiliations:** Dascena, Inc., Houston, TX, United States

**Keywords:** anticoagulant therapy, machine learning, artificial intelligence, clinical trial, stroke prediction

## Abstract

**Background:**

Strokes represent a leading cause of mortality globally. The evolution of developing new therapies is subject to safety and efficacy testing in clinical trials, which operate in a limited timeframe. To maximize the impact of these trials, patient cohorts for whom ischemic stroke is likely during that designated timeframe should be identified. Machine learning may improve upon existing candidate identification methods in order to maximize the impact of clinical trials for stroke prevention and treatment and improve patient safety.

**Methods:**

A retrospective study was performed using 41,970 qualifying patient encounters with ischemic stroke from inpatient visits recorded from over 700 inpatient and ambulatory care sites. Patient data were extracted from electronic health records and used to train and test a gradient boosted machine learning algorithm (MLA) to predict the patients' risk of experiencing ischemic stroke from the period of 1 day up to 1 year following the patient encounter. The primary outcome of interest was the occurrence of ischemic stroke.

**Results:**

After training for optimization, XGBoost obtained a specificity of 0.793, a positive predictive value (PPV) of 0.194, and a negative predictive value (NPV) of 0.985. The MLA further obtained an area under the receiver operating characteristic (AUROC) of 0.88. The Logistic Regression and multilayer perceptron models both achieved AUROCs of 0.862. Among features that significantly impacted the prediction of ischemic stroke were previous stroke history, age, and mean systolic blood pressure.

**Conclusion:**

MLAs have the potential to more accurately predict the near risk of ischemic stroke within a 1-year prediction window for individuals who have been hospitalized. This risk stratification tool can be used to design clinical trials to test stroke prevention treatments in high-risk populations by identifying subjects who would be more likely to benefit from treatment.

## Introduction

As the second most common cause of mortality globally, stroke poses a significant health burden (1). It is associated with long term disabilities, increased healthcare expenditures, and an overall decline in quality of life for individuals who have suffered a stroke (1, 2). In the U.S., over 795,000 strokes occur per year, putting this disease in the top five causes of mortality (3). It is estimated that over $34 billion in healthcare expenditures in the U.S. are directly related to stroke, including lost income, costs associated with management of comorbidities, and use of health services (1, 3). Risk factors for stroke include those that are non-modifiable and modifiable (1). Non-modifiable factors include individual demographics, such as being female, being older than 55, or being a racial-ethnic minority (3–5). Modifiable risk factors include inadequate physical activity, obesity, smoking, and isolation (6, 7).

Ischemic strokes, the most common type of stroke, result from the sudden shortage of blood supply to the brain and account for 80% of strokes in the U.S. and 87% globally (1, 3). Complications can be permanent and pose a range of challenges for stroke survivors, both physically and psychologically (1). For example, a study by Crichton et al. found that nearly 40% of stroke survivors had diagnosed depression following the event and approximately one-third experienced a decline in cognitive abilities (8).

Clinical trials have focused on secondary stroke prevention to influence modifiable risk factors and examine the efficacy of various therapeutic interventions for limiting the recurrence of stroke (9, 10). Anticoagulant therapy has been shown to be an effective tool for primary prevention to reduce stroke risk in patients with comorbidities that put them at a high risk for stroke, such as atrial fibrillation (AF) (11, 12). Given the continued high prevalence of stroke and its lethality, clinical trials are needed to explore the effective use of various therapeutics as both primary and secondary prevention of ischemic strokes in both high risk populations and populations without traditional risk factors. However, clinical trials often stall due to patient attrition or other factors. Per a study by Herrer et al. over one third of all Phase III clinical trials fail due to poor subject selection, resulting in lost expenditures and time for research and development (13).

Artificial intelligence (AI) and machine learning (ML) may serve as tools to supplement the patient selection process for clinical trials by identifying individuals at a high risk for stroke within the window of the study, versus other stroke risk assessments that provide a longer window of prediction. While there has been much progress in the prediction of outcomes of acute stroke using ML-based models (14–17), there is a need for research regarding the utilization of ML tools for the prediction of future stroke. The goal of this study was to examine the ability of ML models to predict an individual's 1-year stroke risk in order to identify individuals for whom preventive interventions, such as anticoagulant therapies, may mitigate this risk. This research may enhance clinical study protocols regarding patient selection, dosage and timing of a study subject's therapy, as well as streamlining the process of patient selection (18).

## Methods

### Data Sources

**Figure 1:**
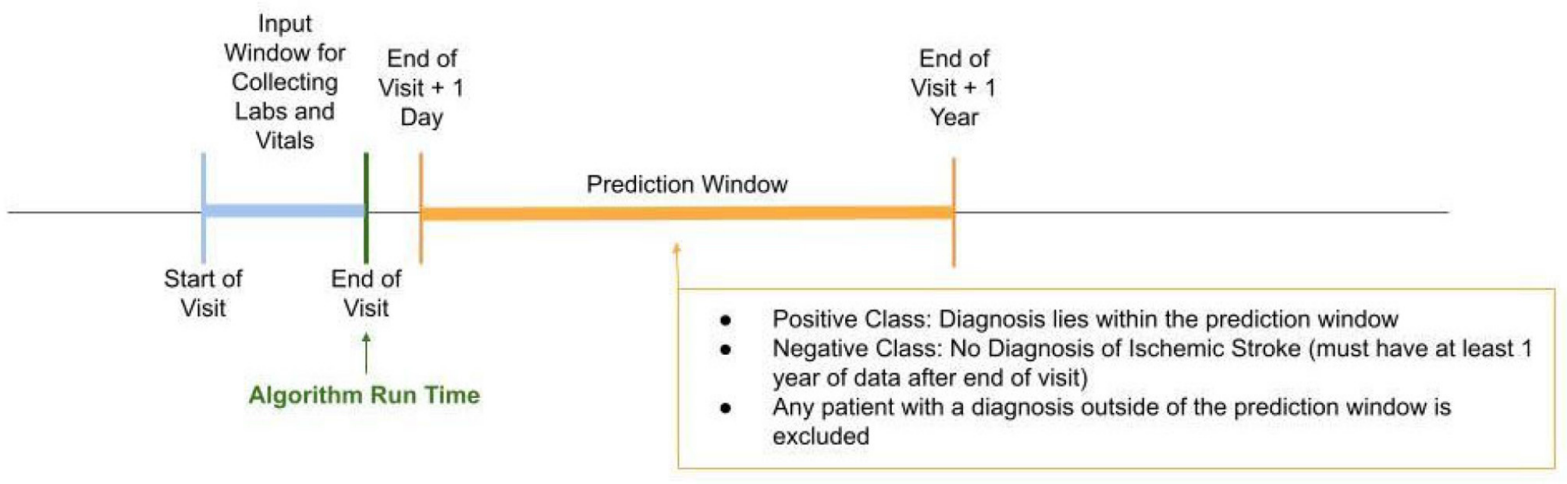
Study design timeline. Patients identified in the positive class according to our gold standard had to have been diagnosed with ischemic stroke within the prediction window, i.e., 1 day after the end of visit to within 1 year from end of visit. The negative class included patients in which no diagnosis of ischemic stroke was identified within the prediction window and they must have had at least 1 year of data after the end of visit.

Data were obtained from a proprietary longitudinal electronic health record (EHR) repository that includes over 700 inpatient and ambulatory care sites located in the U.S. Encounter level data were extracted from individuals between January 2017 and December 2020 (Figure 1). Having had these prior encounters ensured that there was comparison data for these patients in the EHR system. Patient data became eligible for analysis at the patient's second encounter within the same hospital system in either the intensive care unit (ICU) or inpatient wards. Inputs for the analysis included patient demographics, diagnoses, and medication usage both at the time of the first inpatient encounter as well as any prior medication usage recorded in the EHR during the data collection period. Data were collected passively, and to comply with the Health Insurance Portability and Accountability Act (HIPAA), data were de-identified to maintain patient privacy. As data were de-identified, this project did not constitute research using human subjects and approval was not required.

### Patient Selection

Patients who experienced an ischemic stroke between 1 day to 1 year after their first inpatient encounter were identified using international classification of diseases (ICD) codes within EHRs to indicate stroke (Table 1). All patients who had an inpatient encounter, did not meet the criteria for ischemic stroke, and who did not meet the hemorrhagic stroke exclusion criteria were considered to be the negative class (Table 1, Supplementary Table S1). The minimum and maximum timeline for the input window for collecting laboratory and vital measurements was between 24 h and 1,000 h during the patient's length of stay. We excluded encounters that did not fall within that window. Wherever applicable, we used summary statistics (mean value, standard deviation, and last measurement) of collected feature data at any time within the visits. Patients with characteristics indicative of high risk of hemorrhagic stroke at the first encounter were excluded to further improve the ability of the algorithm to only identify patients at risk of ischemic stroke. This software feature has the potential to serve as a tool to reduce the risk of enrolling patients who are at risk for hemorrhagic stroke as opposed to ischemic stroke, as anticoagulant therapy may increase the risk of hemorrhagic stroke (19). Risk factors for hemorrhagic stroke included patients who were given anticoagulants during the first inpatient encounter, had a surgery within 30 days of their first encounter, had a gastrointestinal bleed, amniotic embolism, intracranial hemorrhage, ulcers, and/or had a high risk of falling, or were pregnant. Patients with coagulopathy were also excluded, as these patients were unlikely to be suitable candidates for a clinical trial.

**Table 1:** Inclusion and exclusion details. International classification of diseases version 10 (ICD-10) codes were used to determine inclusion of ischemic stroke patients.

**Diagnosis of ischemic stroke** • I63, H34.1, H34.2
**Exclusion criteria** • Fall risk • Bleeding risk (as determined by prior diagnosis of ICH, GI bleed, history of ulcers, coagulopathy) • Recent surgery (surgery in the last 30 d) • Patients is on anticoagulants • Patient had a diagnosis of an amniotic fluid embolism • Patient is pregnant • No recorded diagnoses or no recorded procedures

Algorithm inputs included demographic information, medical history, and clinical and laboratory data which were identified from EHRs by the use of clinical measurements, ICD codes, procedure data, medicine (self-administered prescription or in-hospital medication) data, and other patient data. An analysis of the correlation between features used in the study was performed and if two features had a very high magnitude of correlation (>0.8), then one of the features was removed. This included the following sets of features: male and female; antihypertensive medication and antidiabetic medication; white blood cell count and platelet count, weight and body mass index (BMI). The list of features used in the model is presented in Table 2.

**Table 2:** Features used in the model.

**Demographic information** Age Sex Race (African American, Asian, Caucasian, Unknown or Other Race) Ethnicity (Hispanic, Not Hispanic) **Clinical measurements** Systolic blood pressure Diastolic blood pressure Heart rate Temperature Body Mass Index (BMI) **Medications** Antihypertensive medication	**Laboratory measurements** Red blood cell (RBC) Hemoglobin Platelets Blood urea nitrogen (BUN) Potassium Glucose Creatinine **Medical history** Atrial fibrillation Congestive heart failure Diabetes Hypertension Vascular diseases Stroke Current smoker

### Machine Learning Model

This research utilized a gradient boosting decision tree classifier to predict ischemic stroke within a year. The Extreme Gradient Boosting (XGBoost v1.3.3) method in Python (v3.6.13) (20–24) was used to implement the decision tree model (25). In this method, multiple trees are generated based on the values of the various input features and a prediction score is generated by combining the results from various trees. During training, future decision trees are constructed with the goal of minimizing the error calculated in previous iterations of tree building. This allows the model to construct targeted trees which optimize the accuracy of the final output. The training process iteratively determines the best variables (and respective thresholds) that can be used to differentiate which patients will have an ischemic stroke within 12 months, and which patients will not. The result of this process is a decision tree that uses a patient's data to predict if they are likely to have a stroke. In handling missing data, we did not include features that had a missing rate of >50%. Furthermore, the XGBoost model was also chosen as it is particularly robust in handling missing data (26, 27) and often outperforms simpler ML models (22, 23). Supplementary Figure S3A shows the missingness of non-categorical features that were used as inputs.

No more than five branching levels were permitted in each tree in the final model. The XGBoost parameter for learning rate was set to 0.2 with no more than 100 total trees to avoid a computational burden. Patients were assigned one of the two groups (predicted ischemic stroke or not predicted ischemic stroke) based on whether or not the final score from the model exceeds a predefined threshold.

Other hyperparameters of the model including the learning rate and the total number trees were selected using a cross-validated grid search. To ensure that model overfitting did not occur, a hyperparameter to prevent iterative tree-addition was built into the training algorithm and optimized across this hyperparameter through the process of 3-fold cross-validation. Another parameter “scale_pos_weight” was introduced and set to a value equivalent to the ratio of negative class examples to positive class examples in order to tackle the imbalance in the dataset. This parameter was optimized as it is useful for unbalanced classes in that it controls the balance of positive and negative weights. This was followed by further optimization of hyperparameters across a sparse parameter grid and cross-validation across a grid search to ensure that an optimal combination of candidate hyperparameters was included in the algorithm.

The final XGBoost model was calibrated post training using the method of isotonic regression (28). Calibration was implemented using the scikit learn package in Python (23). When a model is well-calibrated, the probability associated with the predicted label reflects the likelihood of the correctness of the actual label (29). The reliability curves showing the true probability vs. the predicted probability of the XGBoost model before and after calibration are presented in the Supplementary Figure S4.

### Statistical Analysis

Model performance was determined using a 80-20 train-test split assessed through area under the receiver operating characteristic (AUROC), equivalent to the c-statistic. We reported performance of the model on the test data and an additional external validation dataset (see Supplementary Information). The external validation data comes from a healthcare site and patients separate from those included during model training and testing. The performance of the model against the comparator, the CHA_2_DS_2_-VASc Score (Congestive heart failure, Hypertension, Age > 75, Diabetes Mellitus, Prior Stroke or transient ischemic attack (TIA) or thromboembolism, Vascular disease, Age 65–74 years, Sex category), was assessed by comparing the AUROCs of the model against the comparator on the 20% hold out test set. The 95% confidence intervals of the AUROC curves were calculated by bootstrapping the AUROC curves. The CHA_2_DS_2_-VASc Score was compared in a binary manner (low risk vs. high risk) rather than using risk stratification.

## Results

**Figure 2:**
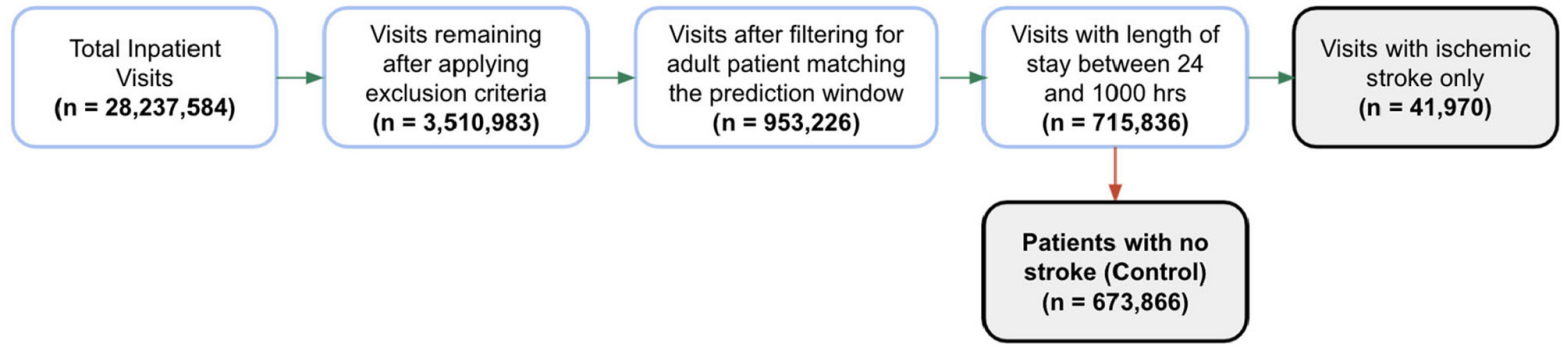
Patient encounter inclusion diagram. Initially, more than 28 million inpatient visits were included in the analysis, then patient encounters were filtered by the exclusion criteria and the prediction window requirements. Forty-one thousand nine hundred seventy patients were identified as positive for ischemic stroke based on our gold standard. The prevalence of ischemic stroke encounters was 5.9% in the training set, 5.8% in the hold-out test set and 6.7% in the external validation set.

In total, 28 million inpatient encounters were initially included in our analysis and 715,836 adult patients were included after applying exclusion criteria and the prediction window condition requirements (Figure 2). Of these encounters, 41,970 patients were identified as positive for ischemic stroke based on our gold standard and 673,866 patients with no stroke diagnosis were classified as the control group. The external validation set consisted of 813,107 total inpatient visits, 56,143 of which were included after applying exclusion filters. Of the 56,143 encounters in the external validation set, 3,790 were identified as positive for ischemic stroke and 52,353 remained in the control group.

Patients who experienced an ischemic stroke were, on average, likely to be older and were more likely to have hypertension, a history or stroke, diabetes or cardiovascular comorbidities (Tables 3, 4).

**Table 3:** Demographic information for the study population sample in the training and testing of the algorithm.

**Demographic information**		**Positive (*N* = 41,970)**	**Negative (*N* = 673,866)**	***P*-value**
Age	18–40	1,705 (4.1%)	163,566 (24.3%)	< 0.0001
	40–60	10,620 (25.3%)	205,509 (30.5%)	< 0.0001
	60–75	15,489 (36.9%)	191,351 (28.4%)	< 0.0001
	75–100	14,156 (33.7%)	113,440 (16.8%)	< 0.0001
Sex	Male	21,499 (51.2%)	307,425 (45.6%)	< 0.0001
	Female	20,397 (48.6%)	364,875 (54.1%)	< 0.0001
	Unknown sex	74 (0.2%)	1,566 (0.2%)	0.0204
Race	African American	7,193 (17.1%)	88,415 (13.1%)	< 0.0001
	Asian	569 (1.4%)	7,050 (1.0%)	< 0.0001
	Caucasian	31,189 (74.3%)	530,059 (78.7%)	< 0.0001
	Unknown or other race	3,019 (7.2%)	48,342 (7.2%)	0.8841
Ethnicity	Hispanic	2,600 (6.2%)	41,696 (6.2%)	0.9501
	Non-hispanic	36,946 (88.0%)	587,308 (87.2%)	0.1747
	Unknown ethnicity	2,424 (5.8%)	44,862 (6.7%)	< 0.0001
Comorbidities	Atrial fibrillation	6,879 (16.4%)	44,382 (6.6%)	< 0.0001
	Diabetes mellitus	15,902 (37.9%)	139,044 (20.6%)	< 0.0001
	Congestive heart failure	8,235 (19.6%)	59,028 (8.8%)	< 0.0001
	History of stroke	24,693 (58.8%)	38,066 (5.6%)	< 0.0001
	Hypertension	31,803 (75.8%)	303,664 (45.1%)	< 0.0001
	Peripheral vascular disease	5,610 (13.4%)	31,981 (4.7%)	< 0.0001
	COPD	8,831 (21.0%)	99,652 (14.8%)	< 0.0001
	Renal (CKD)	9,217 (22.0%)	70,550 (10.5%)	< 0.0001
	Cancer (Leukemia and Lymphoma)	894 (2.1%)	13,946 (2.1%)	0.4069
	Cancer (Solid Tumor)	4,850 (11.6%)	59,280 (8.8%)	< 0.0001

**Table 4:** Demographic information for the study population sample in the external validation dataset.

**Demographic information**		**Positive (*N* = 3,790)**	**Negative (*N* = 52,353)**	***P*-value**
Age	18–40	93 (2.5%)	7,004 (13.4%)	< 0.0001
	40–60	810 (21.4%)	14,972 (28.6%)	< 0.0001
	60–75	1,405 (37.1%)	17,868 (34.1%)	< 0.0001
	75–100	1,482 (39.1%)	12,509 (23.9%)	< 0.0001
Sex	Male	1,858 (49.0%)	23,740 (45.4%)	< 0.0001
	Female	1,932 (51.0%)	28,603 (54.6%)	< 0.0001
	Unknown sex	0 (0.0%)	10 (0.0%)	1
Race	African American	1,060 (28.0%)	10,475 (20.0%)	< 0.0001
	Asian	52 (1.4%)	619 (1.2%)	< 0.0001
	Caucasian	2,551 (67.3%)	39,500 (75.4%)	< 0.0001
	Unknown or other race	127 (3.4%)	1,759 (3.4%)	1
Ethnicity	Hispanic	218 (5.8%)	3,137 (6.0%)	0.5949
	Non-hispanic	3,557 (93.9%)	48,808 (93.2%)	0.7903
	Unknown ethnicity	15 (0.4%)	408 (0.8%)	0.0062
Comorbidities	Atrial fibrillation	839 (22.1%)	7,315 (14.0%)	< 0.0001
	Diabetes mellitus	1,678 (44.3%)	15,709 (30.0%)	< 0.0001
	Congestive heart failure	986 (26.0%)	8,736 (16.7%)	< 0.0001
	History of stroke	2,393 (63.1%)	3,704 (7.1%)	< 0.0001
	Hypertension	3,259 (86.0%)	3,4023 (65.0%)	< 0.0001
	Peripheral Vascular Disease	665 (17.5%)	4,649 (8.9%)	< 0.0001
	COPD	951 (25.1%)	11,759 (22.5%)	< 0.0001
	Renal (CKD)	1,200 (31.7%)	10,054 (19.2%)	< 0.0001
	Cancer (Leukemia and Lymphoma)	71 (1.9%)	964 (1.8%)	0.8514
	Cancer (Solid Tumor)	442 (11.7%)	5,163 (9.9%)	< 0.0001

**Figure 3:**
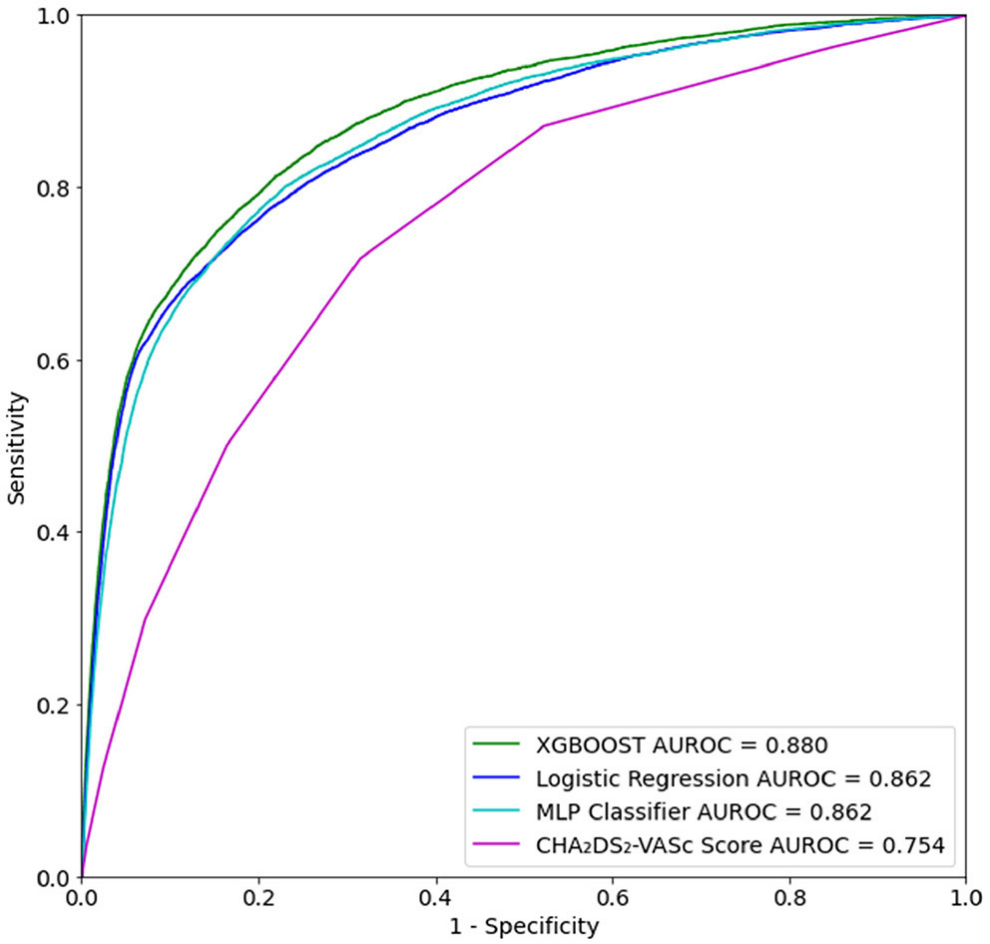
Receiver operating characteristic (ROC) curve for prediction of ischemic stroke for up to 1 year after first inpatient encounter on the test set data.

A total of 41,970 patients with ischemic stroke were included in training and testing of the prediction model. In the test set, XGBoost achieved an area under the receiving operating characteristic (AUROC) curve of 0.880 (95% CI [0.873–0.879]) for prediction of ischemic stroke (Table 5). Logistic Regression and multilayer perceptron (MLP) both achieved comparable AUROCs of 0.862. Though XGBoost and Logistic Regression both performed well, XGBoost may have achieved a slightly higher AUROC for this task because Logistic Regression does not process null values. Logistic Regression imputation of missing data must be done manually, which is not the case for XGBoost. The XGBoost model had a higher specificity than the Logistic Regression model on the hold out test set. Also of note, several prior studies have utilized the XGBoost algorithm to construct models that have superior predictive capacity over existing risk-scoring systems, across a wide range of indications (30–32). The comparator, CHA_2_DS_2_-VASc risk score, achieved an AUROC of 0.7565 (95% CI [0.7531–0.7569]) (Figure 3).

**Table 5:** Performance metrics for XGBoost, logistic regression, and multilayer perceptron (MLP) machine learning algorithms (MLAs) on the testing set and external validation set in comparison to the CHA_2_DS_2_-VASc risk score.

**Hold out test set**								
	**AUROC** **(95% CI)**	**Sensitivity** **(95% CI)**	**Specificity** **(95% CI)**	**PPV** **(95% CI)**	**NPV** **(95% CI)**	**LR+**	**LR-**	**DOR**
XGBoost	0.880 (0.877–0.883)	0.8 (0.791–0.809)	0.793 (0.791–0.796)	0.194 (0.189–0.198)	0.985 (0.984–0.985)	3.87	0.25	15.37
Logistic regression (All Inputs)	0.862 (0.858–0.865)	0.8 (0.791–0.809)	0.754 (0.751–0.756)	0.168 (0.164–0.171)	0.984 (0.983–0.985)	3.25	0.27	12.24
MLP classifier	0.862 (0.863–0.870)	0.8 (0.791–0.809)	0.772 (0.77–0.774)	0.179 (0.175–0.182)	0.984 (0.983–0.985)	3.50	0.26	13.54
CHA_2_DS_2_-VASc Score	0.754 (0.749–0.759)	0.871 (0.864–0.878)	0.479 (0.476–0.481)	0.094 (0.092–0.096)	0.984 (0.983–0.985)	1.67	0.27	6.22
**External validation set**								
XGBoost	0.864 (0.859–0.869)	0.8 (0.787–0.813)	0.749 (0.746–0.753)	0.188 (0.182–0.194)	0.981 (0.98–0.982)	3.19	0.27	11.97
Logistic regression (All Inputs)	0.858 (0.852–0.864)	0.8 (0.787–0.813)	0.745 (0.741–0.749)	0.185 (0.179–0.191)	0.981 (0.98–0.982)	3.14	0.27	11.68
MLP classifier	0.835 (0.830–0.841)	0.8 (0.787–0.813)	0.703 (0.7–0.707)	0.163 (0.158–0.169)	0.98 (0.978–0.981)	2.70	0.28	9.49
CHA_2_DS_2_-VASc Score	0.728 (0.722–0.735)	0.812 (0.8–0.825)	0.519 (0.515–0.523)	0.109 (0.105–0.113)	0.974 (0.973–0.976)	1.69	0.36	4.68

*The testing set included 203,237 total patient encounters with 11,789 patients identified in the positive class. Area under the receiver operating characteristic (AUROC) curve, sensitivity, specificity, positive predictive value (PPV), negative predictive value (NPV), likelihood ratios (LR), and diagnostic odds ratio (DOR) are shown for the MLAs. Supplementary Table S2 shows performance metrics for our XGBoost, logistic regression, and MLP MLAs on the hold out test set and external validation test set using the same inputs as the CHA_2_DS_2_-VASc risk score*.

**Figure 4:**
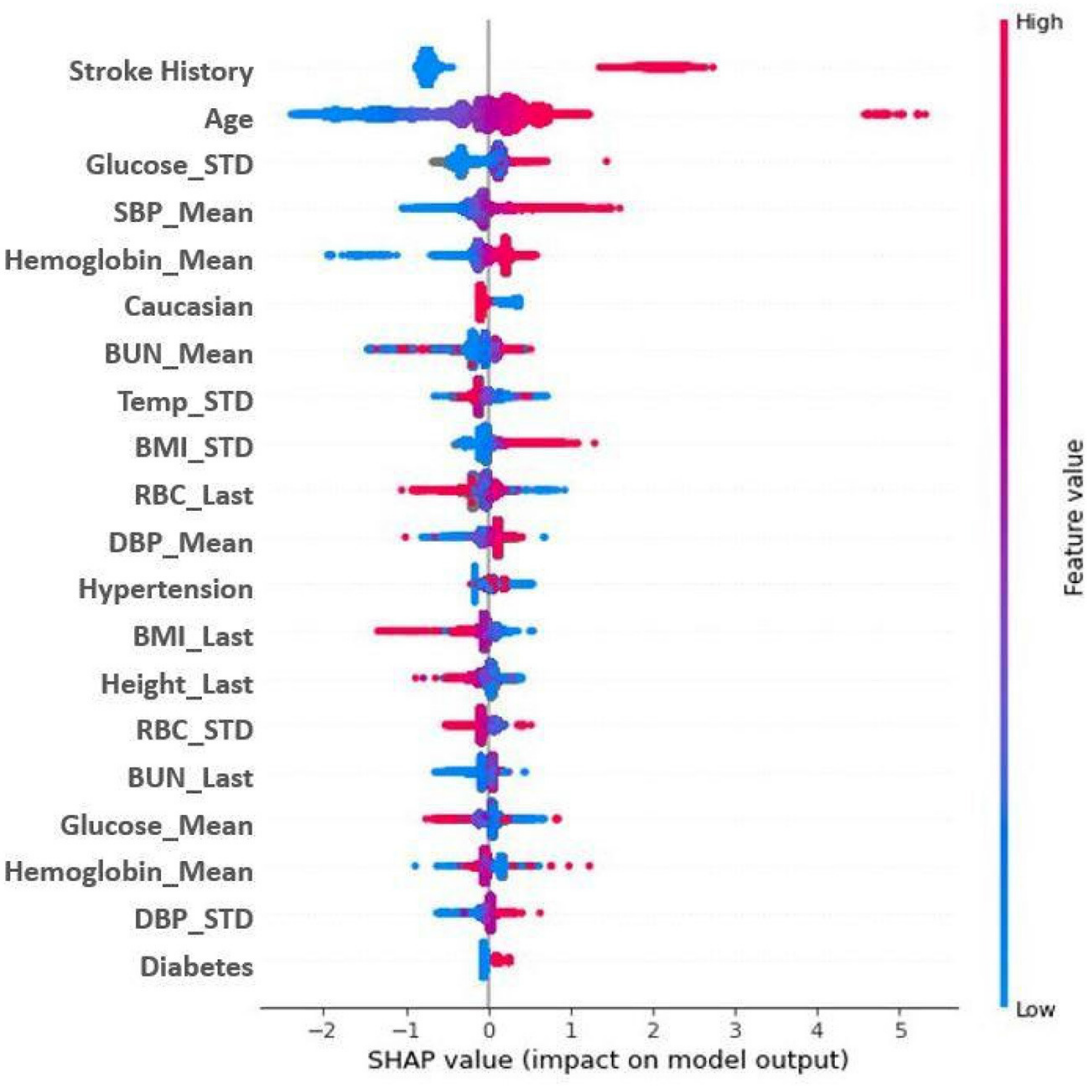
SHAP Plot for model feature importance. Features are ranked in descending order of importance as measured by SHAP values. Red indicates a high feature value; blue indicates a low feature value. Dots to the right are indicative of a higher score; dots to the left a lower score. Mean and STD represent average and standard deviation, respectively. BMI, body mass index; BUN, blood urea nitrogen; CHF, congestive heart failure; DBP, diastolic blood pressure; RBC, red blood cells; SBP, systolic blood pressure; TEMP: temperature.

Feature importance was also assessed using SHAP (SHapley Additive exPlanations: v0.39.0) (33) analysis to determine model features that most significantly impacted ischemic stroke predictions. The SHAP analysis of feature correlation and distribution identified the three most significant features for prediction of ischemic stroke- history of stroke, age, and systolic blood pressure (Figure 4). Important features also identified in the analysis include hypertension, mean hemoglobin, blood urea nitrogen, and temperature. A feature correlation plot is also presented as Supplementary Figure S3B.

## Discussion

### Study Summary

This study describes the development of a machine learning algorithm to accurately predict the onset of ischemic stroke from the period of 1 day up to 1 year following the patient encounter using only data automatically collected from the patient EHR. Although there are existing tools for stroke risk assessment over longer windows of prediction (34, 35), the goal of this study was to develop an MLA tool to aid in the patient selection process for clinical trials by identifying patients at a high risk for ischemic stroke within the time period of a study. The XGBoost algorithm obtained AUROC, PPV, NPV, sensitivity and specificity of 0.864, 0.188, 0.981, 0.800, and 0.749, respectively, on the external test set, indicating the tool's ability to maintain high performance in stroke predictions up to 1 year after an initial inpatient encounter. The use of EHR-based machine learning allows for fast and cost-effective means to identify patients at higher risk of stroke and may potentially improve patient cohorts for clinical trials by accurately predicting shorter term stroke risk. The ability to classify patients as high risk or low risk may guide inclusion and exclusion criteria to ensure that individuals included may have an improved quality of life and decreased incidence of stroke from successful therapies. Importantly, the high negative predictive value of 98.1% indicates the ability of the algorithm to assist researchers to exclude patients who may have otherwise qualified for a clinical trial based on qualitative assessments or patient disclosure of factors that indicated a higher risk for stroke.

The MLA developed and validated in this study outperformed the CHA_2_DS_2_-VASc scoring system, which has been shown to be an effective clinical tool in predicting the 1-year risk of stroke and thromboembolism (TE) in patients both with and without AF (34–36). While the gold standard scoring system that is in wide use for stroke risk assessment is the Framingham Stroke Risk Profile (FSRP) (34, 35), the FSRP tool predicts stroke risk between 5 and 10 years prior to the occurrence of stroke and partially relies on subjective information received directly from patients by a technician-administered questionnaire and a self-administered questionnaire (37). The ability to predict stroke within 1 year may identify patients who have a more immediate risk than those identified in the FRPS, making them viable participants for clinical trials, which occur over limited timeframes. For this study, we chose to use the CHA_2_DS_2_-VASc score as a comparator in order to compare the MLA in this study with a similarly objective risk score that can provide 1-year predictions (36).

### Significant Features

ML methods can provide insight into the importance of individual variables in predicting stroke. The abc (age, biomarker, and clinical history) stroke score was recently shown to provide short-term stroke risk assessment in AF patients (38). In line with these previous findings, history of prior stroke and age were identified as the two most important ML features in our study (Figure 4). Further experimentation was done to examine the performance of the MLAs when stroke history was removed, results for which are presented in Supplementary Table S3, Supplementary Figure S2. Epidemiological studies continue to support the benefits of blood pressure reduction for lowering the risk of stroke (39) as elevated blood pressure levels (>115/75 mm Hg) contribute to almost two-thirds of the global stroke burden. Additionally, both systolic and diastolic blood pressure were ranked among the most important features (top 20), with higher values indicating a higher risk of stroke onset. While diabetes is a known independent risk factor for stroke onset, recent studies have shown that elevated glucose levels and glucose fluctuations (variance) can increase stroke risk, even among individuals without diabetes (40). Similarly, we found that a high variance in glucose level correlated positively with stroke onset. Although the diagnosis of diabetes increased the risk of stroke, the association between mean glucose level (the least important feature on the SHAP plot) and stroke onset was not straightforward. It is plausible that the fluctuation in glucose level is more informative than the mean glucose measurement, particularly in non-diabetic subjects. Fluctuations, as measured by standard deviation, in BMI were positively correlated with stroke risk. These findings are consistent with several previous studies showing that the risk of stroke increases in individuals who lose or gain weight (41). The associations between BMI and stroke risk were inconclusive, possibly reflecting a previously observed weight paradox in stroke outcomes, particularly in the elderly (>75% of our study participants were over 60 years) (42, 43). We also found that a higher potassium concentration was associated with a lower risk of stroke, whereas lower potassium level was associated with a higher stroke risk. These findings are consistent with previous studies reporting associations between low serum potassium and stroke in healthy populations (44) and in adults with hypertension (45).

### Comparison to Other AI Studies

Several studies have examined the use of ML and artificial intelligence (AI) based tools for patient care related to stroke. Ding et al. broadly discuss the role of AI and ML in stroke care and its implications for future stroke management (46). This includes the use of AI to analyze electrocardiogram and ultrasound data for risk stratification and projection of stroke outcomes in patients with known risk factors and to aid with stroke diagnosis using imaging data (46). Sailasya et al. describe the performance of six classification-based MLAs to predict stroke, with the decision-tree model yielding the lowest performance and the Naïve Bayes model yielding the best performance (receiver operating curves 0.66 and 0.82, respectively) (47). A 2019 study by Li et al. examined the use of ML for the purpose of filling in gaps in data that were collected as part of China's national stroke screening and prevention program (48). Two of their models identified an additional ≈5,400 high risk individuals who would not have met the country's standard risk criteria as being high risk. This study indicates the potential for ML to aid with patient selection for clinical trials by identifying individuals who are truly high risk. Patients who have been diagnosed with ischemic stroke are typically only treated with intravenous (IV) thrombolytics if they are within the 4.5 h window of the onset of symptoms (49, 50). However, nearly 25% of patients with acute stroke are unaware of the time of onset of symptoms and are therefore excluded from IV thrombolytic treatment (51, 52). In an effort to determine the time of onset of acute ischemic stroke, Lee et al. applied ML methods on multiparametric MRI scans of patients diagnosed with stroke to retrospectively estimate the time of onset of symptoms (53). This could potentially assist clinicians with determining the best treatment options for patients as well as selecting appropriate candidates for clinical trials for thrombolytics. Ni et al. have suggested that the use of ML may streamline the process of patient selection for clinical trials (18). Ni developed a machine learning algorithm to compare its effectiveness with standard procedures for subject screening and selection for a clinical trial. The results of the study indicated a 34% reduction in time spent by clinical staff for patient recruitment when using the algorithm (18).

### Study Limitations

This study has several limitations. First, the performance of the stroke prediction algorithm was not assessed in prospective settings due to the retrospective nature of the study. To determine how clinicians may respond to predictions of stroke risk, prospective validation is necessary. Prospective validation is also required to determine the extent to which algorithm predictions may affect resource allocation or patient outcomes. Second, stroke risk factors were identified solely via EHR data and healthcare providers may not properly code stroke risk factors or relevant inputs in the EHR (54). Previous studies have reported limited accuracy associated with the ICD-9 stroke codes in identifying ischemic strokes (55, 56). However, ICD-10 stroke codes, as used in this study, are more specific; for instance, ICD-10 codes specify the hemorrhage locations and distinguish between thrombotic and embolic ischemic stroke. Moreover, recent studies have validated the performance of ICD-10 codes for identifying acute ischemic stroke (57). Finally, it is important to note that while the CHA_2_DS_2_-VASc score is a widely-used clinical risk scoring tool for predicting stroke in AF patients (36, 58–60), the cohort utilized in the current study included both AF and non-AF patients. Although the CHA_2_DS_2_-VASc score has been validated for use in non-AF patients, and several clinical studies that have demonstrated the effectiveness of the CHA_2_DS_2_-VASc score in predicting stroke incidence in non-AF patients (61–64), these validation studies are all based on retrospective datasets. The incidence of stroke was predicted by the combination of a large number of EHR features, including several vital signs. While the variation of individual vital signs and lab measures within the normal range are not informative for disease prediction, the ML algorithm can use the variation of a large number of variables to capture a latent pattern for disease prediction. Nevertheless, the biological basis for the contribution of individual vital signs to the ML prediction model is not readily interpretable.

## Conclusion

Clinical trials ensure the safety and efficacy of therapeutics as they transition from development to human testing. However, the success of these measures rely upon a well-identified study cohort. The machine learning algorithm presented in this paper can be successfully utilized to more accurately identify patient cohorts at risk for ischemic stroke within 1 year that are appropriate candidates for anticoagulant therapy studies. This may enable more effective clinical trials of potential ischemic stroke preventative therapies.

## Data Availability Statement

The data analyzed in this study was obtained from a proprietary longitudinal electronic health record (EHR) repository that includes over 700 inpatient and ambulatory care sites located in the U.S. Requests to access the processed data and statistical information should be directed to Qingqing Mao, qmao@dascena.com.

## Author Contributions

RD, QM, and JC contributed to conception and design of the study. JM, YE, and LR assembled the dataset, performed the experiments, and performed the statistical analysis. JM, YE, LR, GB, SS, and AG-S wrote the manuscript. All authors contributed to the article and approved the submitted version.

## Conflict of Interest

All authors are or were employed by Dascena, Inc. (Houston, Texas, U.S.A).

## Publisher's Note

All claims expressed in this article are solely those of the authors and do not necessarily represent those of their affiliated organizations, or those of the publisher, the editors and the reviewers. Any product that may be evaluated in this article, or claim that may be made by its manufacturer, is not guaranteed or endorsed by the publisher.
