# Supplementary material for: Enriching the Study Population for Ischemic Stroke Therapeutic Trials Using a Machine Learning Algorithm
Source: Front Neurol. 2022 Jan 25;12:784250. doi: 10.3389/fneur.2021.784250 (PMC8823366; doi:10.3389/fneur.2021.784250)
Supplement: Supplementary file 1 [file Table_1.pdf]

Supplementary Material

Supplementary Table S1. International classification of diseases version 10 (ICD-10) codes used in the analysis for diagnosis, exclusion criteria, and identification of comorbidities.

Diagnosis	ICD-10 codes
**Ischemic Stroke**	I63, H34.1, H34.2
**Exclusion Criteria**	
**Bleeding Risk**	I60.9, I62.1, I62.0, IG2.9, K92.0, K92.1, K92.2, K25.0, K25.1, K25.2, K25.3, K25.4, K25.5, K25.6, K25.7, K25.9, K56.60, K56.699, D66, D67, D68.1, D68.2, D68.0, D68.311, D68.312, D68.318, D65, D68.32, D68.4, D68.8, D68.9
**Amniotic Fluid Embolism**	O88.111, O88.112, O88.113
**Pregnant**	Z33, Z34, Z36, Z37, Z3A, O09, O10, O11, O12, O13, O14, O15, O16, O20, O21, O22, O23, O24, O25, O26, O28, O29, O30, O31, O32, O33, O34, O35, O36, O40, O41, O42, O43, O44, O45, O46, O47, O48, O60, O61, O62, O63, O64, O65, O66, O67, O68, O69, O70, O71, O72, O73, O74, O75, O76, O77, O80, O82, O88, O98.01, O98.02, O98.11, O98.21, O98.22, O98.31, O98.32, O98.41. O98.42, O98.51, O98.52, O98.61, O98.62, O98.71, O98.72, O98.81, O98.82, O98.91, O98.8=92, O99.01, O99.02, O99.11, O99.12, O99.210, O99.211, O99.213, O99.214, O99.280, O99.281, O99.282, O99.283, O99.284, O99.310, O99.311, O99.312, O99.313, O99.314 O99.320 O99.321 O99.322 O99.323 O99.324 O99.330 O99.331 O99.332 O99.333 O99.334 O99.340 O99.341 O99.342 O99.343 O99.344 O99.350 O99.351 O99.352 O99.353 O99.354 O99.41 O99.42 O99.51 O99.52 O99.61 O99.62 O99.71 O99.72 O99.73 O99.810 O99.814 O99.820 O99.824 O99.830 O99.834 O99.840 O99.841 O99.842 O99.843 O99.844 O99.891 O99.892 O9A.11 O9A.12 O9A.21 O9A.22 O9A.31 O9A.32 O9A.41 O9A.42 O9A.51 O9A.52
**Comorbidities**	
**Sepsis**	A40, A41, R65.2, T81.12,T81.44, O85, O86.04
**Cardiovascular (Hypertension)**	I10, I11, I12, I13, I15, O10, O11
**Cardiovascular (Heart Failure)**	I50, I11.0, I13.0, I13.2
**Cardiovascular (Peripheral Vascular Disease)**	I73.9, I73.89, I70.2, I70.3, I70.4, I70.5, I70.6, I70.7, I70.9, E08.5, E09.5, E10.5, E11.5, E13.5
**Renal**	N18, E08.22, E09.22, E10.22, E11.22, E13.22
**Liver**	K70, K71, K72, K73, K74, K75, K76, K77
**Cancer**	Z85, Z17, C00, C01, C02, C03, C05, C06, C07, C08, C09, C10, C11, C12, C13, C14, C15, C16, C17, C18, C19, C20, C21, C22, C23, C24, C25, C26, C31, C32, C33, C34, C37, C38, C39, C40, C41, C43, C44, C4A, C45, C46, C47, C48, C49, C50, C51, C52, C53, C54, C55, C56, C57, C58, C60, C61, C62, C63, C64, C65, C66, C67, C68, C69, C70, C71, C72, C73, C74, C75,

	C76, C77, C80, C7A, C7B, C81, C82, C83, C84, C85, C86, C88, C90, C91, C92, C93, C94, C96
**Diabetes**	E08, E09, E10, E11, E13, O24.0, O24.1, O24.3, O24.8, O24.9, Z79.4
**Pneumonia**	J12, J13, J14, J15, J16, J17, J18, J69, A22.2, B01.2, B37.1, J95.891

Supplementary Table S2. Performance metrics for our XGBoost and logistic regression MLAs on the hold out test set and external validation test set **using the same inputs as** the CHA_2DS_2-VASc risk score.

**Hold out test set**								
	**AUROC (95% CI)**	**Sensitivity (95% CI)**	**Specificity (95% CI)**	**PPV (95% CI)**	**NPV (95% CI)**	**LR+**	**LR-**	**DOR**
**Xgboost**	0.876 (0.873 - 0.879)	0.8 (0.792 - 0.808)	0.787 (0.785 - 0.788)	0.182 (0.179 - 0.186)	0.985 (0.984 - 0.986)	3.75	0.25	14.75
**Logistic Regression (CHA2DS2VASc Inputs)**	0.847 (0.844 - 0.85)	0.8 (0.792 - 0.808)	0.709 (0.707 - 0.711)	(0.138 - 0.143)	(0.983 - 0.984)	2.75	0.28	11.58
**CHA_2DS_2-VASc Score**	0.756 (0.753 - 0.76)	0.871 (0.865 - 0.877)	0.491 (0.489 - 0.494)	0.092 (0.091 - 0.094)	0.985 (0.984 - 0.985)	1.71	0.26	6.52
**External test set**								
**Xgboost**	0.866 (0.861 - 0.871	0.8 (0.788 - 0.812)	0.764 (0.761 - 0.768)	0.196 (0.19 - 0.202)	0.196 (0.19 - 0.202)	3.40	0.26	12.98
**Logistic Regression (CHA2DS2VASc Inputs)**	0.850 (0.844 - 0.856)	0.8 (0.788 - 0.812)	0.736 (0.733 - 0.74)	0.178 (0.173 - 0.184)	0.981 (0.98 - 0.982)	3.02	0.27	11.16
**CHA_2DS_2-VASc Score**	0.731 (0.725 - 0.737	0.805 (0.793 - 0.817)	0.532 (0.527 - 0.536)	0.11 (0.106 - 0.113)	0.974 (0.973 - 0.976)	1.72	0.37	4.68

Supplementary Table S3. Performance metrics for our XGBoost, logistic regression, and MLP machine MLAs in comparison to the CHA_2DS_2-VASc risk score on the hold out test set and external test set excluding patients with stroke history.

Hold out Test Set								
	**AUROC (95% CI)**	**Sensitivity (95% CI)**	**Specificity (95% CI)**	**PPV (95% CI)**	**NPV (95% CI)**	**LR+**	**LR-**	**DOR**
**Xgboost**	0.781 (0.775 - 0.787)	0.8 (0.787 - 0.813)	0.622 (0.619 - 0.625)	0.054 (0.052 - 0.056)	0.991 (0.991 - 0.992)	2.12	0.32	6.58
**Logistic Regression (All Inputs)**	0.747 (0.741 - 0.754)	0.8 (0.787 - 0.813)	0.558 (0.556 - 0.561)	0.047 (0.045 - 0.049)	0.990 (0.990 - 0.991)	1.81	0.36	5.06
**MLP Classifier**	0.736 (0.728 - 0.743)	0.8 (0.787 - 0.813)	0.518 (0.515 - 0.52)	0.043 (0.042 - 0.045)	0.990 (0.989 - 0.990)	1.66	0.39	4.30
**Logistic Regression (CHA2DS2VASc Inputs)**	0.708 (0.702 - 0.715)	0.803 (0.789 - 0.816)	0.497 (0.494 - 0.499)	0.042 (0.04 - 0.043)	0.989 (0.989 - 0.99)	1.59	0.40	4.01
**CHA_2DS_2-VASc Score**	0.657 (0.650 - 0.665)	0.912 (0.903 - 0.922)	0.166 (0.164 - 0.168)	0.029 (0.028 - 0.03)	0.986 (0.984 - 0.987)	1.09	0.53	2.07
**External Validation Set**								
**Xgboost**	0.725 (0.715 - 0.735)	0.8 (0.779 - 0.821)	0.531 (0.527 - 0.536)	0.047 (0.044 - 0.049)	0.989 (0.988 - 0.991)	1.71	0.38	4.54
**Logistic Regression (All Inputs)**	0.725 (0.714 - 0.735)	0.8 (0.779 - 0.821)	0.529 (0.525 - 0.533)	0.047 (0.044 - 0.049)	0.989 (0.988 - 0.991)	1.70	0.38	4.50
**MLP Classifier**	0.670 (0.658 - 0.681)	0.8 (0.779 - 0.821)	0.438 (0.433 - 0.442)	0.039 (0.037 - 0.042)	0.987 (0.986 - 0.989)	1.42	0.46	3.12
**Logistic Regression (CHA2DS2VASc Inputs)**	0.690 (0.679 - 0.701)	0.8 (0.779 - 0.821)	0.471 (0.466 - 0.475)	0.042 (0.039 - 0.044)	0.988 (0.987 - 0.989)	1.51	0.42	3.56
**CHA_2DS_2-VASc Score**	0.660 (0.649 - 0.672)	0.868 (0.85 - 0.885)	0.323 (0.319 - 0.327)	0.036 (0.034 - 0.037)	0.988 (0.987 - 0.99)	1.28	0.41	3.13
