# Supplementary material for: Enriching the Study Population for Ischemic Stroke Therapeutic Trials Using a Machine Learning Algorithm
Source: Front Neurol. 2022 Jan 25;12:784250. doi: 10.3389/fneur.2021.784250 (PMC8823366; doi:10.3389/fneur.2021.784250)
Supplement: Supplementary file 2 [file Image_1.pdf]

TABLES

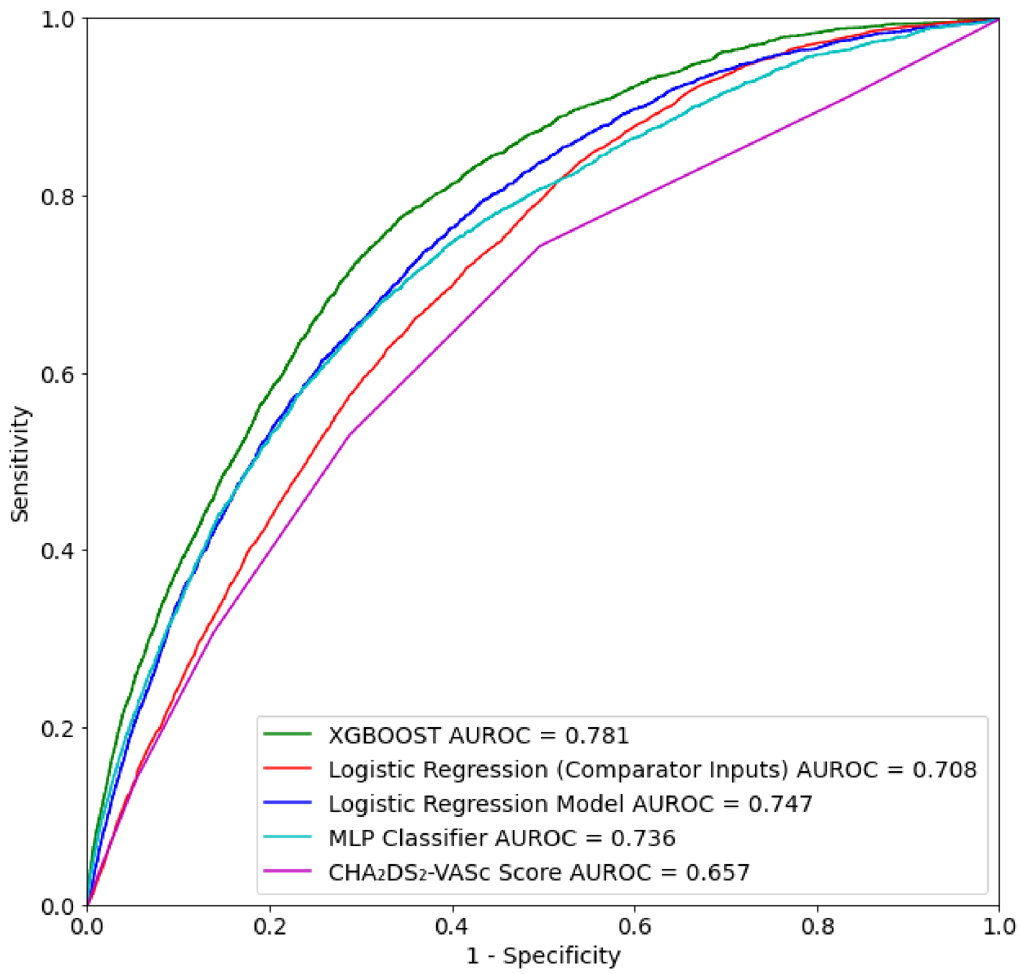

Supplementary Figure S1. Receiver operating characteristic (ROC) curve for prediction of ischemic stroke for up to one year after first inpatient encounter using the hold out test set excluding patients with stroke history.

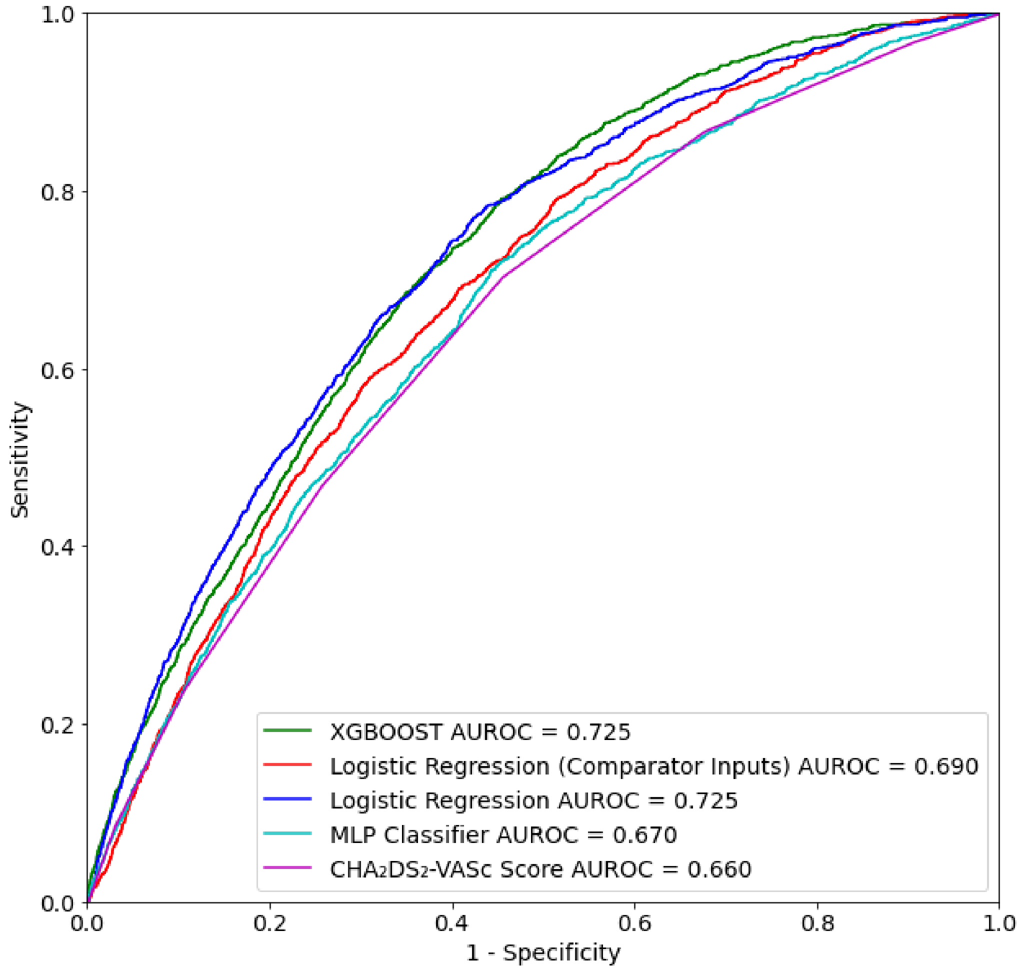

Supplementary Figure S2. Receiver operating characteristic (ROC) curve for prediction of ischemic stroke for up to one year after first inpatient encounter using the external test set excluding patients with stroke history.

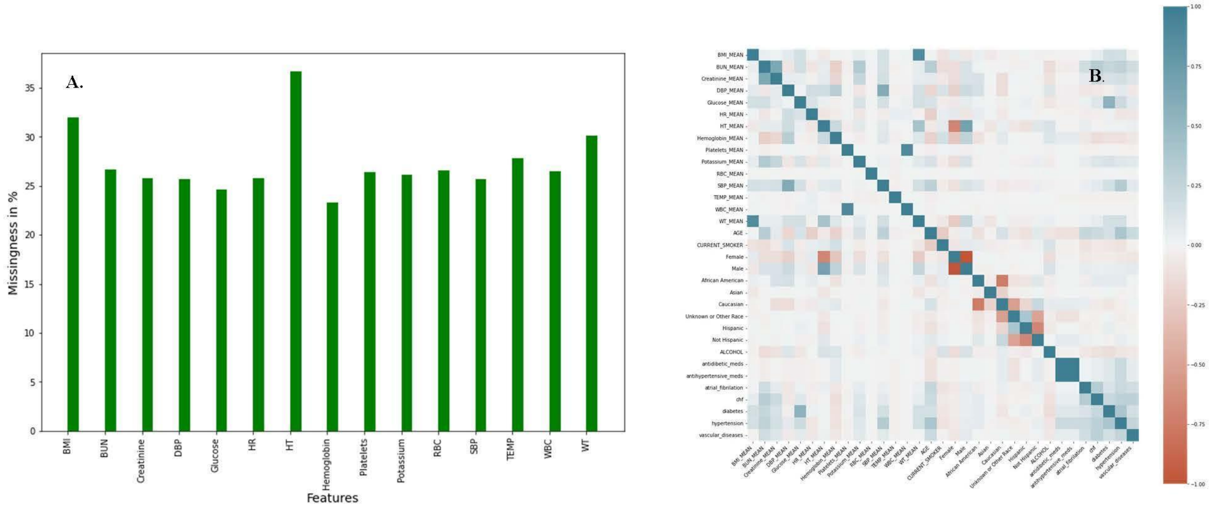

Supplementary Figure S3. A. Missingness of non-categorical features that were used as inputs; B. Feature correlation plot.

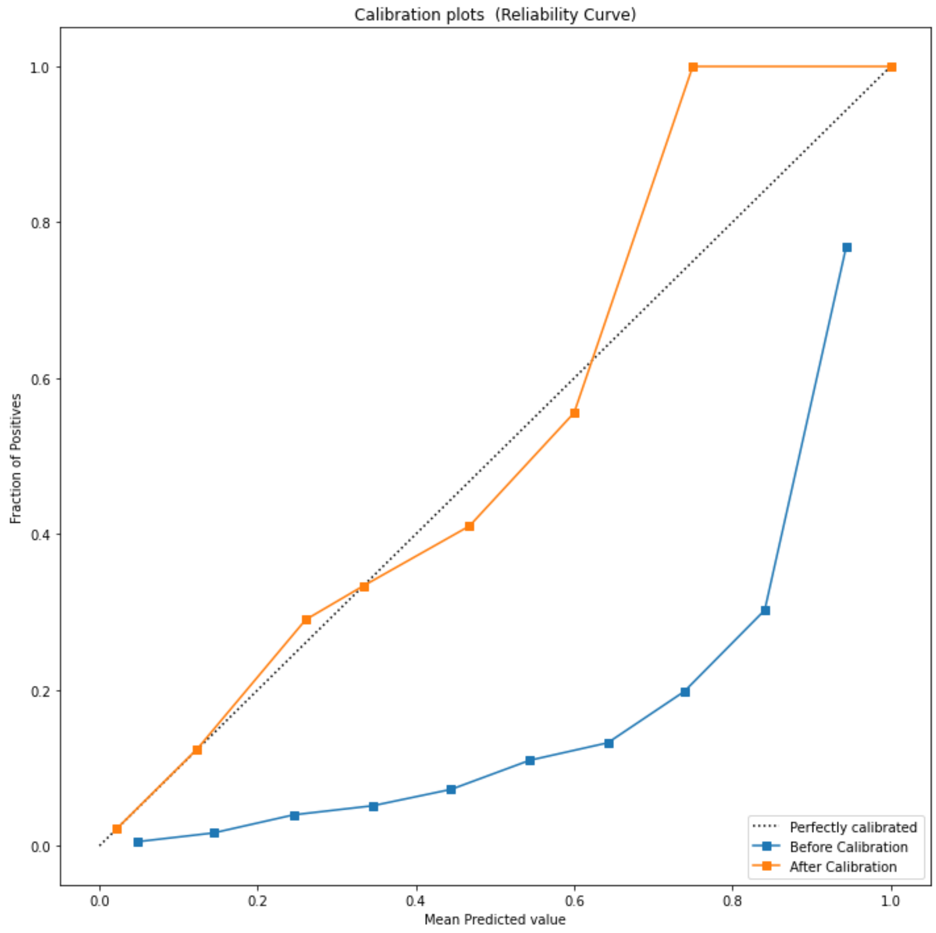

Supplementary Figure S4: True probability vs. the predicted probability of the XGBoost model before and after calibration.
